# Establishment of a Therapeutic Anti-Pan HLA-Class II Monoclonal Antibody That Directly Induces Lymphoma Cell Death via Large Pore Formation

**DOI:** 10.1371/journal.pone.0150496

**Published:** 2016-03-30

**Authors:** Shuji Matsuoka, Yasuyuki Ishii, Atsuhito Nakao, Masaaki Abe, Naomi Ohtsuji, Shuji Momose, Hui Jin, Hisashi Arase, Koichi Sugimoto, Yusuke Nakauchi, Hiroshi Masutani, Michiyuki Maeda, Hideo Yagita, Norio Komatsu, Okio Hino

**Affiliations:** 1 Department of Pathology and Oncology, Juntendo University School of Medicine, Tokyo, 113–8421, Japan; 2 RIKEN Research Center for Allergy and Immunology, Yokohama, 204–0022, Japan; 3 Department of Immunology, Faculty of Medicine, Yamanashi University, Yamanashi, 409–3898, Japan; 4 Department of Immunochemistry, WPI Immunology Frontier Research Center, Osaka University, Osaka, 565–0871, Japan; 5 Department of Hematology and Oncology, JR Tokyo Hospital, Tokyo, 151–8523, Japan; 6 Division of Stem Cell Therapy, Center for Stem Cell Biology and Regenerative Medicine, Institute of Medical Science, The University of Tokyo, Tokyo, 108–8639, Japan; 7 Department of Cell Biology, Institute for Virus Research, Kyoto University, Kyoto, 606–8507, Japan; 8 Department of Immunology, Juntendo University School of Medicine, Tokyo, 113–8421, Japan; 9 Division of Hematology, Department of Internal Medicine, Juntendo University School of Medicine, Tokyo, 113–8421, Japan; European Institute of Oncology, ITALY

## Abstract

To develop a new therapeutic monoclonal Antibody (mAb) for Hodgkin lymphoma (HL), we immunized a BALB/c mouse with live HL cell lines, alternating between two HL cell lines. After hybridization, we screened the hybridoma clones by assessing direct cytotoxicity against a HL cell line not used for immunization. We developed this strategy for establishing mAb to reduce the risk of obtaining clonotypic mAb specific for single HL cell line. A newly established mouse anti-human mAb (4713) triggered cytoskeleton-dependent, but complement- and caspase-independent, cell death in HL cell lines, Burkitt lymphoma cell lines, and advanced adult T-cell leukemia cell lines. Intravenous injection of mAb 4713 in tumor-bearing SCID mice improved survival significantly. mAb 4713 was revealed to be a mouse anti-human pan-HLA class II mAb. Treatment with this mAb induced the formation of large pores on the surface of target lymphoma cells within 30 min. This finding suggests that the cell death process induced by this anti-pan HLA-class II mAb may involve the same death signals stimulated by a cytolytic anti-pan MHC class I mAb that also induces large pore formation. This multifaceted study supports the therapeutic potential of mAb 4713 for various forms of lymphoma.

## Introduction

Monoclonal antibodies (mAbs) have dramatically improved the treatment of lymphoma. This is particularly true for non-Hodgkin lymphoma (NHL), which can be treated with rituximab (anti-CD20 mAb) [1,2]. However, rituximab only improves clinical outcome in combination with chemotherapy, and a subset of the patients become rituximab-resistant after repetitive treatments [3]. However, there is currently no mAb therapy available for Hodgkin’s disease. Radiation therapy, chemotherapy, and combination therapy have been used to treat Hodgkin lymphoma (HL) for many years with relatively good outcomes [4]. But these therapies are associated with the risks of sterility, secondary leukemia, and therapy-related myelodysplastic syndrome [5]. In addition, adult T-cell leukemia (ATL) is a very aggressive form of malignancy caused by T-cell transformation induced by human T-lymphotropic virus type 1 (HTLV-1) infection [6]. The prognosis of ATL is very poor, with a median survival time of only 24 months despite the current therapies [7]. Irradiation and chemotherapy are not effective against ATL. Therefore, there is an urgent need for new therapeutic agents addressing HL and ATL.

The principle behind our cytolytic anti-lymphoma mAb therapy is based on observations made in animal studies. Unlike nude or SCID mice, normal strains of mice inoculated with live malignant human cells survive and reject the inoculated cells [8]. During the first or second challenge, the malignant cells are primarily killed by NK cells and CD8+ T cells, or ingested by macrophages. However, during the course of repeated inoculations with malignant cells, mouse lymphocytes generate antibodies against the malignant human cells. These antibodies may constitute as major contributors to the rejection of malignant cells due to their efficacy in killing target cells. This hypothesis served as the basis for experiments aimed at establishing cytolytic anti-lymphoma mAbs.

Most therapeutic mAbs against cell surface molecules exert their effects mainly through immunological mechanisms, including complement-dependent cytotoxicity (CDC) and antibody-dependent cellular cytotoxicity (ADCC). In addition to indirectly inducing Fc-dependent cell death, several mAbs also directly induce programmed cell death [9–13]. Hybridoma clones were selected based on the direct cytotoxicity of their supernatants to HL lymphoma cells. During the screening process, we ignored ADCC and CDC because they may be ineffective in lymphoma/leukemia patients immunocompromised by radiation, chemotherapy and the malignant disease itself. Consequently, we identified an anti-pan HLA class II mAb with a direct cytotoxic effect on lymphoma/leukemia cells, including HL, NHL, and advanced ATL cells.

The aim of the present study was to investigate the cytotoxic activity of this newly established anti-pan HLA class II mAb in several types of lymphoma/leukemia cell lines, both *in vitro* and *in vivo*. We also compared the mechanism of cell death induced by this antibody and the anti-pan MHC class I mAb we previously established [11–13]. The cell death induced by anti-pan MHC class I and class II mAbs was cytoskeleton-dependent but caspase- and complement-independent. We demonstrate that cytolytic anti-MHC class I and II mAbs directly induce the death of lymphoma cells via a giant pore formation on their surface with the shared death mechanism.

## Materials and Methods

### Ethics

This study has been per performed according to the principles of Helsinki, and approved by the Ethical committee at Juntendo University School of Medicine (permission ID number 14–195).

### Mice and cells

The BALB/c and C.B-17/ICR-SCID mice [14] at 8 weeks of age were obtained from Japan SLC Inc. (Hamamatsu, Japan) and CLEA Japan Inc. (Tokyo, Japan), respectively. All mice were maintained under specific pathogen-free conditions. The Ethics Review Committee for Animal Experimentation of Juntendo University Faculty of Medicine approved all animal experiments (Project Number 727). Mice were euthanized using sodium pentobarbital, and appropriate efforts were made to minimize suffering.

Human peripheral mononuclear cells were collected from healthy adult volunteers. Informed, written consent was obtained from all study participants. The documents of participant consent are preserved in each participant’s medical records. Peripheral blood lymphocytes were isolated from consenting healthy blood donors via centrifugation over Histopaque (Sigma Chemical Co., St. Louis, MO). Human embryonic kidney 293T cells and human lymphoma cell lines (L428, L540, KMH-2, Raji, Daudi, MOLT-4, C1R, HT, Jurkat, SU-DHL-1, K562, NKL, NK-YS, and HANK-1) were purchased from the American Type Culture Collection (Manassas, VA), the German Collection of Microorganisms and Cell Cultures/DSM (Braunschweig, Germany), or the Japan Cancer Research Resources Bank (Osaka, Japan). The HTLV-1-infected IL-2-dependent cells (ATL-26c, ATL-72/2, and ED-40515) were established from samples obtained from consenting ATL patients by cell culture in the presence of IL-2. The HTLV-1-infected IL-2-independent cells (ATL-2, ATL-16, and ED40815) were established from long-term cultures of IL-2-dependent cells. Each set of IL-2-dependent and IL-2-independent cells had the same clonal origin, which was confirmed by examining the rearrangement of T-cell receptor-γ gene and HTLV-1 proviral integration sites [15].

All lymphoma cells and ATL cell lines were cultured in RPMI 1640 or DMEM medium containing 10% heat-inactivated fetal calf serum (FCS) and antibiotics (100 U/ml penicillin and 100 μg/ml streptomycin) at 37°C, in the presence of 5% CO_2_. The IL-2-dependent HTLV-1-infected T cells and NK lymphoma cell lines were maintained by adding IL-2 (7.5 ng/ml; PeproTech EC; London, UK) to the culture medium.

### Transfectants

The 293T cell line was transiently transfected with pME18S expression plasmid containing HLA-DP, HLA-DQ, or HLA-DR cDNA and pMx-GFP expression plasmid using polyethylenimine (Polysciences; Tokyo, Japan). Two days after transfection, their expression of HLA-DP or HLA-DR on the transfectants was confirmed by FACS using HLA-DP/HLA-DR mAb HL-40 (EXBIO; Tokyo, Japan) or HLA-DQ-specific mAb HLA-DQ1 (BioLegend; Tokyo, Japan).

### Reagents and antibodies

Latrunculin B was purchased from Biomol Research Labs (PA, USA). Z-VAD-FMK and Z-Asp-DCB were purchased from the Peptide Institute Inc. (Osaka, Japan), and cathepsin inhibitor III (Z-FG-HNO-BzOME) from Calbiochem (Tokyo, Japan). IM-54 was purchased from Santa Cruz Biotechnology (UK). Necrostatin-1 was purchased from Enzo Biochem Inc. (NY). EDTA, forskolin, cytochalasin D, 4,5-dihydroxy-1, 3-benzenedisulfonic acid (Tiron), Alexa 488-conjugated rat anti-mouse Ig, and mouse IgG were purchased from Sigma-Aldrich. Anti-asialo GM1 was purchased from Wako Pure Chemical Industries (Osaka, Japan). Anti-Fas mAb was purchased from MBL (Nagoya, Japan). Gout anti-rabbit IgG (H+L) antibody Alexa 488 conjugate was purchased from Thermo Fisher Science (MA).

### Establishment of mAb 4713

A BALB/c mouse was immunized intraperitoneally every 2 weeks for 3 months with live HL cells. The injections were alternated between L428 and KM-H2 cells to establish mAbs reactive to common molecules on the surface of HL cell lines. Three days after the last immunization, mouse spleen cells were fused with P3U1-nonproducing myeloma cells using the polyethylene glycol method, and the resulting hybridomas were selected using hypoxanthine–aminopterin–thymidine (HAT) culture medium (Corning Cellgro; VA, USA). Hybridomas producing antibodies showing direct cytolytic activity against a 3^rd^ HL cell line, L540, were selected based on the trypan blue dye exclusion test. This immunizing and screening method was adopted to establish mAbs with direct cytotoxic effects on multiple lymphoma cell lines, and to prevent the establishment of mAbs clonotypically reactive to a single cell line. We identified one mAb (4713) that consistently killed HL cell lines directly.

### Flow cytometry analysis and cytotoxicity assay

The molecular targets of mAb 4713 were analyzed by incubating cells with mouse mAb 4713, followed by Alexa 488-conjugated rat anti-mouse IgG on ice, and then subjecting the cells to flow cytometry analysis using a BD LSRFortessa cell analyzer (BD Biosciences).

The cytotoxic activity of mAb 4713 was assessed by incubating a mixture of target cells (2 × 10^6^ cells/ml) in RPMI medium containing 2% decomplemented (56°C, 30 min) FCS and the mAb (3 μg/ml) at 37°C for 2 h (unless stated otherwise). The percentage of lysed cells was determined in triplicate based on trypan blue dye exclusion or PI staining, followed by FACS on the BD LSRFortessa cell analyzer. As the results obtained through PI staining were nearly identical to those obtained by trypan blue dye exclusion, only the dye exclusion results are presented.

Potential inhibitors of mAb 4713 cytotoxicity were added to the cell cultures either 1 h before (z-VAD-FMK, z-Asp-DCB, LY294002, cytochalasin D, and latrunculin B) or 2 h before (wortmannin) the addition of mAb 4713. Sodium azide, EDTA, and cytochalasin D were added to the assay medium during the cytolytic assay to test the effects of these reagents. The concentration of each reagent was optimized during preliminary experiments based on methods described previously [11, 12, 16].

### Tumor xenograft experiments

Five C.B-17/ICR-SCID mice were injected intraperitoneally with rabbit anti-asialo GM1 (Wako Pure Chemicals) to deplete the NK cells. After 24 h, they received an intravenous injection of Raji cells (5 × 10^6^) suspended in 200 μl of PBS. Five days, or 5 and 12 days, after inoculation of Raji cells, the mice were injected intravenously with mAb 4713 (1 μg/mouse).

### Affinity chromatography

Purified mAb 4713 (5 mg) was immobilized using HiTrap NHS-activated HP (1 mL; GE Healthcare Inc.), according to the manufacturer’s protocol. A total of 1 × 10^8^ cells were incubated (5 min; 4°C) in PBS (pH 7) containing 1% (w/w) Nonidet P-40 (Wako Chemical, Japan) and a protease inhibitor cocktail (Roche, Inc.). After centrifugation, the supernatant was recovered and applied on the 4713 mAb-HiTrap. After washing the column with 5 volumes of lysis buffer, the bound proteins were eluted with 0.1% (w/w) glycine–HCl (pH 2.7), and neutralized with 1 M Tris-HCl (pH 9.0).

### Western blot analysis

Protein samples were separated by SDS-PAGE and transferred to a PVDF membrane. After treatment with Pierce Fast Blocking Buffer (Pierce Biotechnology Inc., Tokyo, Japan), the membrane was incubated with buffer containing mAb 4713 (1 μg/mL), followed by a horseradish peroxidase-conjugated secondary antibody. The membrane was treated with enhanced chemiluminescent (ECL) reagent (Amersham; Tokyo, Japan), and the reactive protein bands were visualized with a Fujifilm image analyzer.

### Scanning electron microscopy

Samples of L428 cells were incubated with mAb 4713 (37°C; 15 or 30 min), and then washed and resuspended in PBS containing 2% FCS. The cell suspension was fixed by adding 0.1 volume of 1% glutaraldehyde in 0.1 M cacodylate buffer (pH 7.3), and incubating the samples at 4°C for 2 h. The fixed cells were washed with 0.1 M cacodylate buffer, post-fixed in 1% OsO_4_ (1 h; 4°C), dehydrated in approximately 50%–100% ethanol, substituted with t-butyl alcohol, dried at −10°C under a vacuum, and observed with a high resolution scanning electron microscope (S-900; Hitachi LTD, Tokyo).

### Statistical analysis

All experiments were conducted in triplicate, and the data were expressed as mean ± SD. The resulting mean values were <10% SD. Statistical analyses were performed using SPSS 14.0 software (IBM, NY). The data sets were compared by Student’s *t* tests, and P values <0.05 were considered significant.

## Results

### The cytotoxic activity of mAb 4713 against multiple types of lymphoma cells

**Fig 1:**
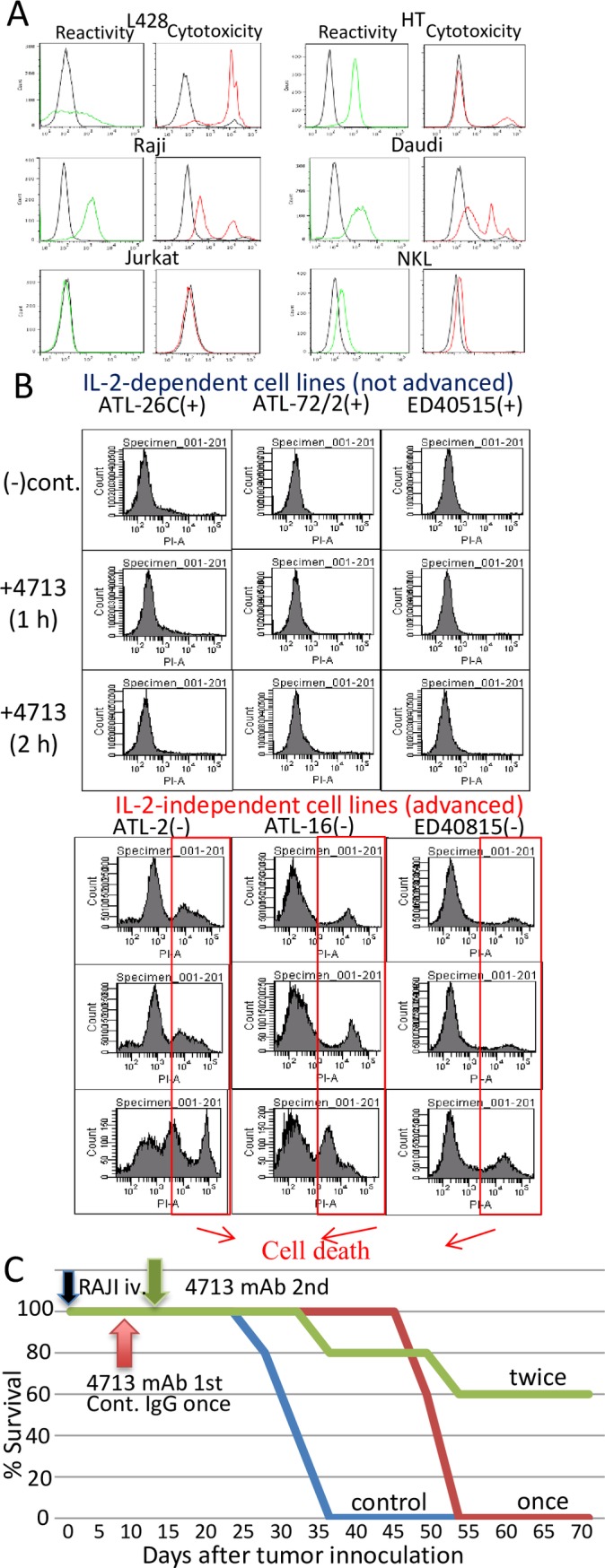
Reactivity and cytotoxic activity of mAb 4713 against lymphoma cell lines. (A) Flow cytometry analysis of mAb 4713 reactivity analyzed by incubating different lymphoma cell lines with mAb 4713 (4°C; 2 h), followed by Alexa 488-conjugated rat anti-mouse Igs (green histogram). Cytotoxicity was analyzed based on propidium iodide (PI) staining of dead cells (red histogram). (B) Cytotoxic effects of mAb 4713 on IL-2-dependent or IL-2-independent ATL cell lines analyzed by PI staining after incubation with mAb 4713 (37°C; 1 or 2 h). Similar data were obtained in two independent experiments. (C) Kaplan–Meier survival analysis of mAb 4713-treated C.B-17/ICR-SCID mice bearing Raji lymphoma xenografts. The Raji-injected SCID mice (n = 5) were treated with mAb 4713 once (red line) or twice (brown line), or with control IgG once (green line). **p = 0.01 versus isotype control.

One cloned mAb, named mAb 4713 induced rapid cell death in HD lymphoma cell line, L428, dose-dependently (S2 Fig). The cytolytic activity of mAb 4713 was also tested against various types of lymphoma cells, including HL and NHL cell lines. The cells were incubated with mAb 4713 at 37°C for 2 h (Table 1 upper column). The treatment induced rapid cell lysis in several cell lines. All of the tested HL cell lines showed varying degrees of mortality. Approximately 30% to 90% of the cells were killed within 2 h. Non-HL cells, including Burkitt lymphoma cells, were also killed by mAb 4713. The observed cytotoxicity was independent of serum complements because heating ascitic fluid (56°C; 30 min) did not affect the cytotoxicity (unpublished data), and cell death occurred even under serum-free culture conditions. The sensitivity of lymphoma cell lines to mAb 4713-induced cell death and cell-surface binding of mAb 4713 were confirmed by flow cytometry. Dead cells were stained with propidium iodide (PI) (Fig 1A). Whereas mAb 4713 stained NK lymphoma cell lines, they were not sensitive to mAb 4713-induced cell death (Table 1 upper column). Even the NK lymphoma cell line (NKL) with a high expression level of the target molecules was not susceptible to mAb 4713-induced cell death (Fig 1A). Thus, there was no correlation between the expression level of target molecule and the cell death sensitivity.

**Table 1:** Cytotoxic effects of mAb4713 on lymphoma/leukemia cell lines.

Cell line	Cell type	Reactivity	% killing	Comment
Cytotoxic effects on various lymphoma cell lines				
L428	Hodgkin lymphoma	positive	92 ±2	HLA-DR(+) CD20(+)
L540	Hodgkin lymphoma	positive	40 ±3	HLA-DR(+) CD15(+)
KM-H2	Hodgkin lymphoma	positive	32 ±4	HLA-DR(+) CD58(+)
RAJI	Burkitt lymphoma	positive	58 ±2	HLA-DR(+) CD25(+)
Daudi	Burkitt lymphoma	positive	56 ±3	MHC class I-deficient
C1R	B cell lymphoma	positive	65 ±3	MHC class I-deficient
HT	B lymphoblast	positive	68 ±2	Diffuse mixed lymphoma
Jurkat	T cell lymphoma	negative	0 ±0	HLA-DR(−) Fas(+)
MOLT-4	ALL (T cell)	negative	0 ±2	HLA-DR(−) CD4(+)
SU-DHL-1	diffuse large	negative	0 ±0	HLA-DR(−) CD25(+)
K562	myelogenous	negative	0 ±3	HLA-DR(−) CD25(+)
NKL	NK lymphoma	positive	0 ±1	HLA-DR(+) IL-2-dependent
NKYS	NK lymphoma	positive	0 ±2	HLA-DR(+) IL-2-dependent
HANK-1	NK lymphoma	positive	0 ±0	HLA-DR(+) IL-2-dependent
Cytotoxic effects on ATL cell lines				
ATL-26c	ATL	positive	0 ±3	IL-2-dependent
ATL-72/2	ATL	positive	0 ±3	IL-2-dependent
ED-40515	ATL	positive	0 ±3	IL-2-dependent
ATL-55T	ATL	positive	52±6	IL-2-independent
ATL-2	ATL	positive	73±3	IL-2-independent
ED-40810	ATL	positive	69±4	IL-2-independent

The reactivity of mAb 4713 was determined by FACS analysis as described in Fig 1.

Lymphoma and ATL cells (2 × 10^6^ cells/ml) were incubated with mAb 4713 (3 μg/ml) for 2 h at 37°C. The percentages of dead cells (% killing) were determined based on trypan blue dye exclusion and are indicated as means±SD of triplicated samples.

### The cytotoxic activity of 4713 against ATL cell lines

We tested the cytolytic activity of mAb 4713 against ATL cell lines, which were classified as either non-advanced (IL-2-dependent) or advanced (IL-2-independent) types (15). None of the IL-2-dependent ATL cells tested were killed by mAb 4713. In contrast, all of the IL-2-independent ATL cell lines tested were sensitive to 4713 killing. A total of 50% to 70% of the IL-2-independent ATL cells were killed by 4713 within 2 h (Table 1 lower column and Fig 1B). Similar results were obtained for IL-2-dependent ATL cell lines cultured without IL-2 and for IL-2-independent ATL cell lines cultured with IL-2 (unpublished data), indicating that exogenous IL-2 has no direct effect on mAb 4713-induced cell death in ATL cells. As mAb 4713 was not cytotoxic to NK lymphoma cell lines that depend on supplemental IL-2 for culture maintenance, we conclude that mAb 4713 has cytolytic activity only for lymphoma/leukemia cells that proliferate without exogenous IL-2.

**Fig 2:**
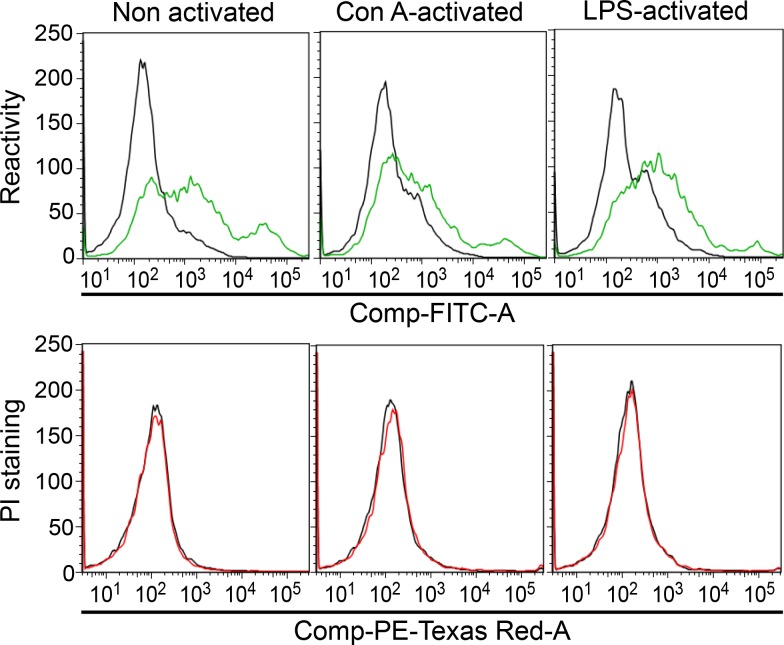
Lack of cytotoxicity of mAb 4713 on lymphocytes from healthy donors. Freshly isolated, or Concanavalin (Con) A- or LPS-activated (12 h), peripheral blood lymphocytes were incubated with mAb 4713 for 2 h, then with Alexa 488-conjugated anti-mouse IgG (green) or propidium iodide (red). Negative controls are shown by black lines.

Normal peripheral blood lymphocytes collected from healthy donors also reacted with mAb 4713, but they were not sensitive to mAb 4713-induced killing, even after Concanavalin (Con) A- or lipopolysaccharide (LPS)-mediated activation (Fig 2). Therefore, mAb 4713 does not damage non-cancerous lymphocytes. Despite its broad spectrum of binding activity, the cytotoxic activity of mAb 4713 was limited to IL-2-independent lymphoma cell lines (Table 1, Figs 1 and 2).

### The anti-tumor activity of mAb 4713 in a tumor-bearing mouse model

The potential therapeutic effect of mAb 4713 against lymphoma was tested *in vivo* using the human tumor xenograft SCID mouse model. To deplete NK cells, the mice were pretreated with anti-asialo-GM1 antisera 1 day before Raji Burkitt lymphoma cells were xenografted. Thereafter, mAb 4713 or control mouse IgG was injected intravenously on day 5, or on day 5 and 12, after injection of the Raji cells. Fig 1C shows that mAb 4713 significantly improved survival, compared with the control IgG-treated mice. On day 70 after treatment, 60% of the mice treated with two doses of mAb 4713 were still alive, whereas all those treated with a single dose were dead (Fig 1C). These data demonstrate that mAb 4713 causes a dose-dependent increase in survival in mice bearing Raji Burkitt's lymphoma xenografts. Although we tried to inoculate HD cells to SCID mouse intensely, any dose of HD cells tested could not be engrafted to SCID mice successfully. Therefore we could not demonstrate the therapeutic effect of mAb 4713 on HD cells in vivo.

### The molecular targets of mAb 4713

**Fig 3:**
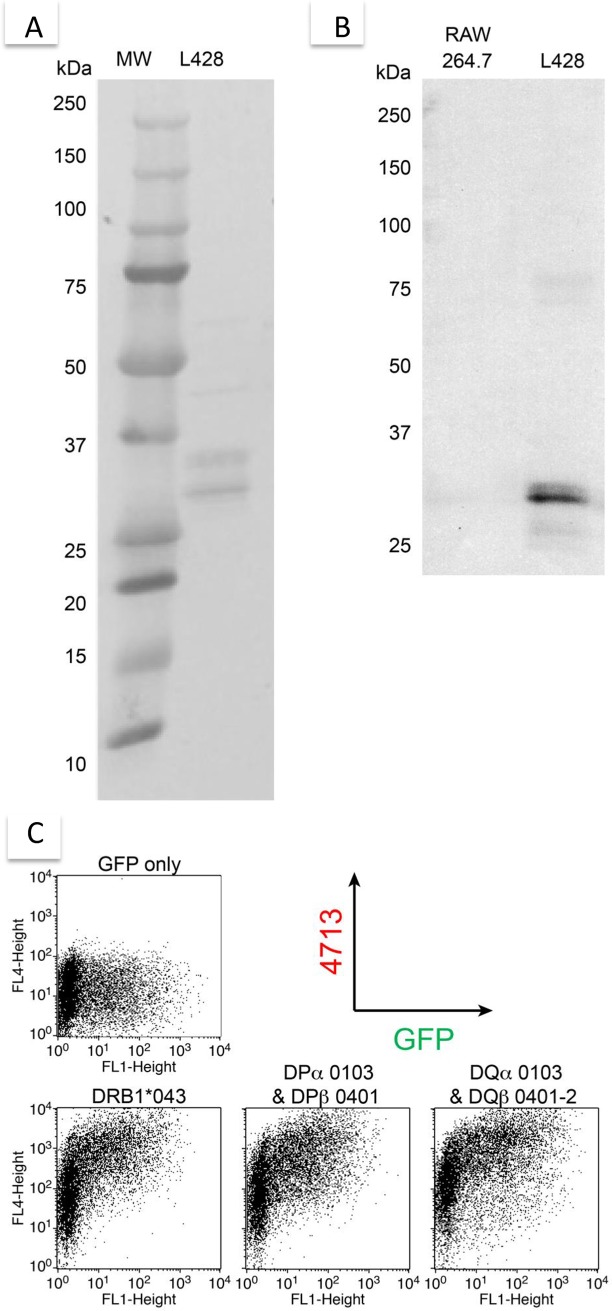
Reactivity of mAb 4713 with HLA class II molecules. (A) Purification of mAb 4713-binding proteins from L428 cell lysate by affinity chromatography using mAb 4713. (B) Western blot analysis of the affinity-purified proteins isolated from RAW264.7 or L427 cell lysate using mAb 4713. (C) Flow cytometry analysis of mAb 4713 reactivity with HLA-DP-, HLA-DQ-, or HLA-DR-transfected 293T cells. Cells were incubated with mAb 4713, followed by PE-conjugated rat anti-mouse Igs.

The molecular targets of mAb 4713 were identified by affinity chromatography using column-bound mAb 4713. Lysates of mAb 4713-sensitive L428 HL cells [17] were applied to the column, and the eluate was recovered with glycine–HCl buffer. Fig 3A shows that mAb 4713 bound to two major proteins of 28 kDa and 32 kDa. They were identified as the HLA-DR α-chain and β -chain by peptide mass fingerprinting analysis. Western blot analysis using mAb 4713 detected only the 28-kDa protein (Fig 3B). Collectively, these data suggest that the molecular target of mAb 4713 is the HLA-DR β-chain. Accordingly, the HLA class II-positive cell lines were reactive to mAb 4713, whereas many of the HLA class II-negative cell lines were not (Table 1, S1 Fig). High HLA class II expression has been reported on ATL cells [18]. Therefore, we tested whether mAb 4713 recognizes other MHC class II antigens, namely HLA-DP, HLA-DQ and HLA-DR. We prepared HLA-DP, HLA-DQ, or HLA-DR transfectants, and analyzed their reactivity with mAb 4713. Flow cytometry indicated that mAb 4713 stained all of the HLA class II-transfected cells, but not the control cells transfected with green fluorescent protein (GFP) alone (Fig 3C). Together, these data suggest that mAb 4713 recognizes an epitope common to all HLA class II β-chains. However, the transfectants were not sensitive to mAb 4713-induced killing. Therefore, the cell surface expression of HLA class II molecules on target cells is essential, but not sufficient, for mAb 4713-induced cell death.

### The mAb 4713-induced cell death signaling pathway

**Fig 4:**
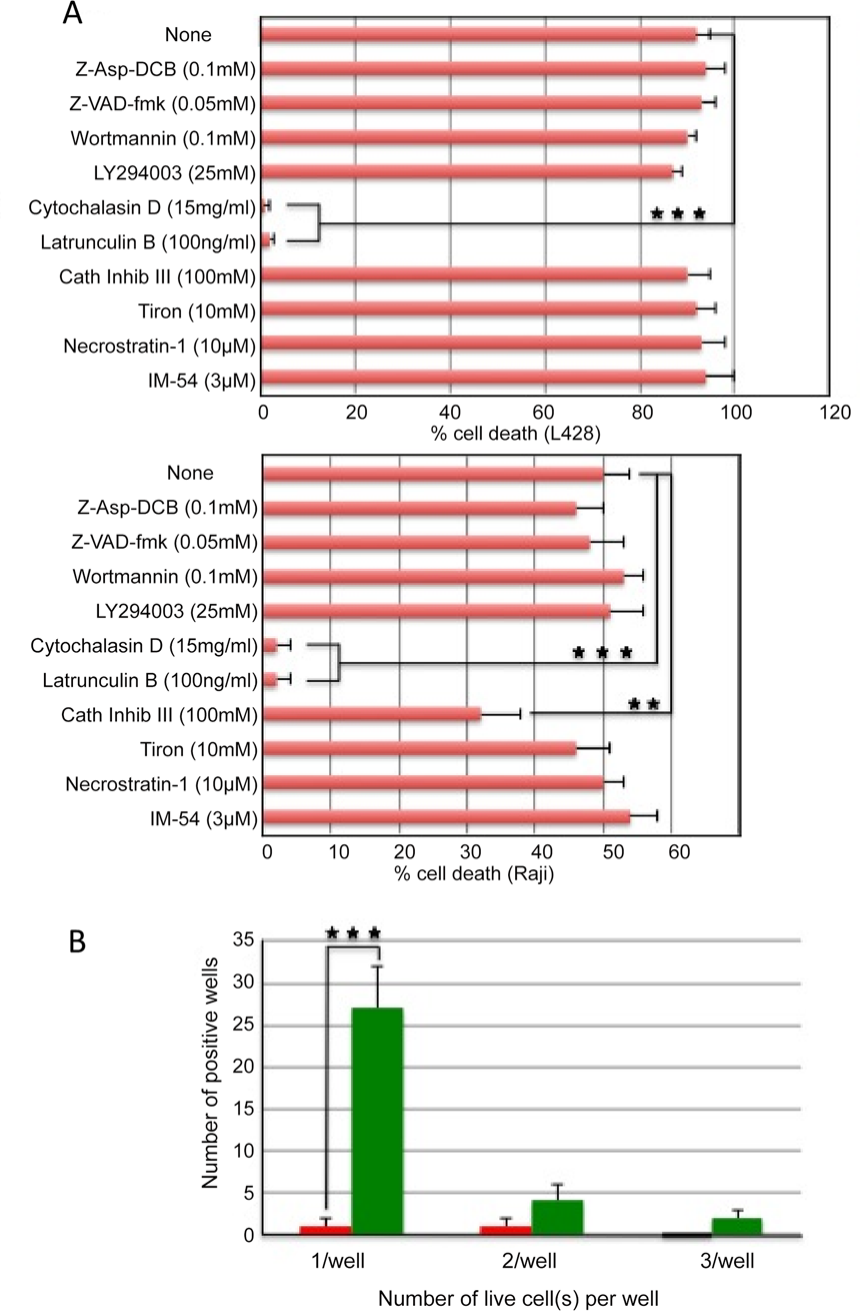
Mechanism of mAb 4713-induced cell death involving the cytoskeleton. (A) Impact of chemical inhibitors on the cytotoxic activity of mAb 4713 against L428 and Raji cells. The inhibitors were added 1 or 2 h before the cytotoxicity assay. The percentage of dead cells was determined by trypan blue dye exclusion. **p = 0.01 versus none. ***p = 0.001 versus none. The agents were caspase inhibitors (z-VAD-FMK and z-Asp-DCB), PI-3 kinase inhibitors (wortmannin and LY294002), cytoskeletal inhibitors (cytochalasin D and latrunculin B), necroptosis inhibitor (Necrostatin-1), necrosis inhibitor (IM-54), a cathepsin inhibitor (Cath inib III), and reactive oxygen species scavenger (Tiron). (B) Limiting dilution experiments in which L428 cells were seeded (0.3 cells/well; 96 wells) with 3 μg/ml mAb 4713 or control IgG. After 2 h, the number of live cells per well was counted. **p = 0.01 versus isotype control. ***p = 0.001 versus control IgG. Each value represents the mean ± SD.

The underlying mechanism of mAb 4713-induced cell death was investigated by testing the effects of several potential inhibitors on the cytotoxic activity of mAb 4713 against L428 HL cell line and Raji Burkitt lymphoma cell line. The caspase inhibitors, z-VAD-FMK [19] and z-Asp-DCB [20], and the PI-3 kinase inhibitors, wortmannin [21] and LY294002 [22], did not inhibit the cytotoxic activity of mAb 4713 (Fig 3A). To confirm the difference between mAb 4713 induced cell death from apoptosis, we performed several experiments. Western blot analysis supported the caspase-independent cell death mechanism. Treatment of Jurkat cells with cytochrome C showed bands of cleaved caspase-3, but treatment of L428 cells with mAb 4713 did not (S3A Fig). Similarly cleaved caspase-3 was not detected in L428 cells treated with mAb 4713 by flow cytometric analysis (S3B Fig). Furthermore, depolarization of mitochondrial membrane was not observed during mAb 4713-induced cell death (S4 Fig). The necroptosis inhibitor, Necrostatin-1 [23], and the necrosis inhibitor, IM-54 [24], also failed to inhibit the cytotoxic activity of mAb 4713 (Fig 4A). Consequently, mAb 4713-induced cell death is not apoptosis, necroptosis, nor necrosis. In contrast, both cytochalasin D, which depolymerizes cytoskeletal actin filaments to actin monomers [25], and latrunculin B, which reduces the monomeric actin pool available for polymerization [26], completely inhibited mAb 4713-induced cell death (Fig 4A). These data are reminiscent of previous findings regarding the cytolytic activity of anti-pan MHC class I mAb against lymphocytes [11, 12]. Therefore, cytolytic anti- pan MHC class I and class II antibodies may use the shared signaling events to induce cell death. We hypothesized that cell death was initiated by a disorganization of cytoskeletal actin filament systems induced by intensive cross-linking between anti-pan MHC mAbs and abundant cell surface MHC molecules because it was reported that Fab fragments of anti-pan MHC mAbs have no cytolytic activity, whereas cross-linking of Fab with anti-mouse Igs reconstituted cytotoxicity in T cells ([11] and our unpublished data].

Type II anti-CD20 mAbs and some anti-HLA-DR mAbs have been reported to induce non-apoptotic cell death in lymphoma and leukemia cells through a reactive oxygen species (ROS)-dependent pathway [16]. The extent of mAb-induced cell death correlated with the generation of ROS mediated by NADPH oxidase. In the case of type II anti-CD20 mAbs, cell death involved lysosomal membrane permeabilization and subsequent release of lysosomal cathepsins into the cytosol to trigger ROS production. In the present study, a cell-permeable ROS scavenger 4,5-dihydroxy-1, 3-benzenedisulfonic acid (Tiron) had no effect on the mAb 4713-induced cell death, suggesting a ROS-independent mechanism (Fig 4A). As a matter of fact, ROS production was not observed after exposure to mAb 4713 (S5 Fig). These results suggest that mAb 4713 induces cell death ROS-independently.

In addition, cathepsin inhibitor III was reported to prevent type II anti-CD20 mAb-induced cytotoxicity in Raji cells [16]. We observed a cell type-dependent response of mAb 4713-induced cell death to cathepsin inhibitor III (Fig 4A). This compound reduced the killing of Raji Burkitt lymphoma cells by 30%, but had no effect on the survival of L428 HL cells. These results suggest that lysosomal cathepsins constitute non-essential participants in the mAb 4713-induced killing.

Several type II anti-CD20 mAbs and an anti-HLA-DR mAb were recently reported to induce caspase and complement-independent non-apoptotic cell death [16, 27]. Therefore, these type II anti-CD20 mAbs may use a similar cell death signals to our anti-pan HLA-class II mAb. However, at least some of the death signals associated with these mAbs are different. For example, type II anti-CD20 mAb-mediated cell death was associated with the induction of homotypic intercellular adhesion [16]. Furthermore, actin redistribution toward cell-to-cell contact area was both critical for cell aggregation and subsequent cell death stimulated by type II anti-CD20 mAb or anti-HLA-DR mAbs [27]. In the present study, limiting dilution experiments were conducted to test whether aggregation and homotypic adhesion are required for the mAb 4713-induced cell death. Target L428 HL cells were seeded (0.3 cells/well; 96 wells) with 3 μg/ml mAb 4713 or control mouse IgG. After 2 h, most wells contained a single live cell, namely 30 wells with IgG and 3 wells with mAb 4713 (Fig 4B). These data show that a single target cell was killed by mAb 4713 in the absence of homotypic adhesion or aggregation.

### Light and electron microscopy findings

**Fig 5:**
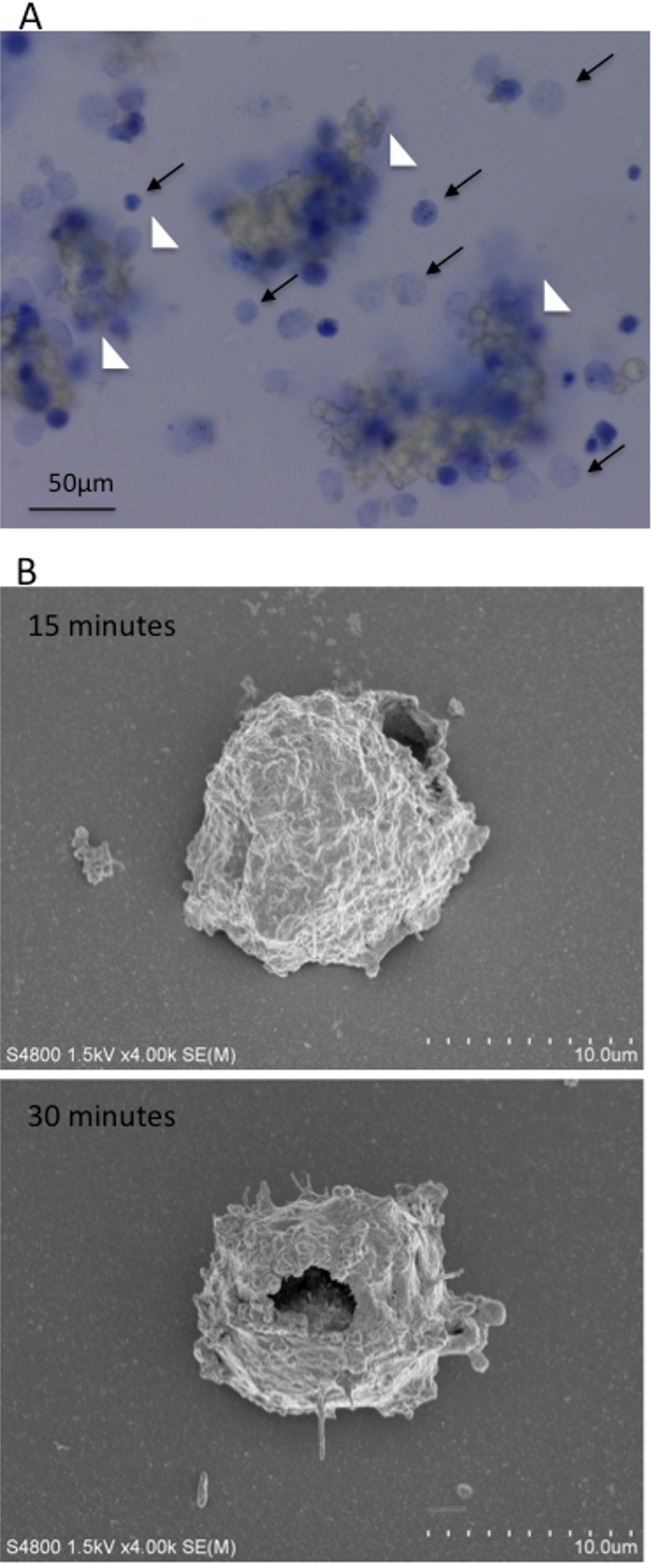
Microscopy analysis of mAb 4713-induced single cell death shows giant pores. (A) Light microscopy image showing that the incubation of L428 cells with mAb 4713 caused rapid aggregation and death of single cells after 30 min, as shown by trypan blue exclusion staining. ←, single cell death; ∆, aggregation of cells. Bar: 50 μm (B) Scanning electron microscopy image showing the formation of giant pores on L428 Hodgkin lymphoma cells after a 15 or 30 min incubation with mAb 4713.

The mechanism of mAb 4713-induced cell death was visualized using different microscopy approaches. First, light microscopic examination confirmed the occurrence of single cell death, in the absence of aggregation, after a 30-min incubation of L428 HL cells with mAb 4713 (Fig 5A). Second, scanning electron microscopy revealed the formation of giant pores at the surface of L428 HL target cells during the early phase of mAb 4713-induced killing (Fig 5B). The giant pores (approximately 3 μm) appeared as early as 15–30 min after the addition of mAb 4713 to the culture medium. This finding is distinct from those observed during apoptotic or necrotic cell death.

## Discussion

We designed an immunizing and screening method to establish a mAb reactive to common molecules on the surface of HL cell lines. This method identified an anti-pan HLA class II mAb (mAb 4713) with direct cytotoxic effect on HL, NHL, and advanced ATL cells, but inactive on peripheral blood lymphocytes from healthy donors. The therapeutic potential of anti-pan HLA class II mAb 4713 was clearly demonstrated *in vivo* in the human tumor xenograft SCID mouse model, even after single treatment. Therefore, this mAb presents tremendous therapeutic potentials for a variety of leukemias/lymphomas.

Some mAbs against MHC class I and class II molecules were shown to induce apoptotic or non-apoptotic cell death in lymphoma cells and activated lymphocytes [11–13, 28–31]. Their ligation to cell surface MHC molecules rapidly and directly induces complement- and caspase-independent cell death. Whereas MHC class I molecules are abundantly expressed on almost all somatic cells, MHC class II molecules are confined to various antigen-presenting cells of the immune system (B cells, activated T cells, macrophages, and dendritic cells). It is noteworthy that non-cancerous and resting lymphocytes are resistant to the cytolytic effects of mAbs targeting MHC molecules. Thus, cell death induced by anti-MHC mAbs is selective for lymphoma cells and activated lymphocytes.

Mouse anti-pan HLA class II mAb 4713 directly induced non-apoptotic cell death in lymphoma cells. Giant pores were detected at the surface of the target cells within 30 min of mAb 4713 exposure. We previously reported the formation of similar giant pores on mouse T cells treated with rat anti-mouse MHC class I mAb RE2 (S6 Fig)[11]. This antibody recognized all strains of mice, except MHC class I-deficient mice. Furthermore, mAb RE2 prevented the binding of anti-H-2K^k^ and anti-H-2D^k^ mAbs to the surface of H-2k haplotype cells. These data showed that mAb RE2 recognizes pan-MHC class I molecules. We also demonstrated that mAb RE2 induces direct cell death by cytoskeleton-dependent, but caspase- and complement-independent, mechanisms [11, 12], as in the case of mAb 4713. In fact, cell death induced by anti-pan HLA class II mAb 4713 and anti-pan MHC class I mAb RE2 shares similar chemical sensitivity profiles. In both cases, cytoskeletal inhibitors (cytochalasin D and latrunculin B) reduced cell killing by >90%, whereas caspase inhibitors (Z-VAD-fmk and Z-Asp-DCB) and PI-3 kinase inhibitors (wortmannin and LY294002) had no effect [12]. Collectively, these data suggest that the antibody-mediated ligation of MHC class I or MHC class II molecules may induce cell death in lymphoma cells through common signaling machineries. We named the cell death induced by cytolytic anti-pan MHC antibodies as anapocosis. Anapoco means holes in Japanese.

The anti-CD20 mAb rituximab shows improved clinical outcomes in patients with non-Hodgkin lymphoma when used in combination with chemotherapy. Moreover, an anti-CC chemokine receptor 4 (CCR4) mAb has been developed as a novel therapeutic agent for ATL patients [32,33]. However, a substantial proportion of patients remain to suffer from relapses and acquire resistance to these mAb therapies. In addition, some patients with lymphoma/leukemia are immunocompromised. Therefore, the ability of the anti-pan HLA class II mAb 4713 to induce complement- and ADCC-independent cell death may present a clinical advantage over conventional therapeutic mAbs for leukemia/lymphoma.

## Supporting Information

S1 Fig Expression of HLA-DR on various cell lines. Various cell lines indicate in Table 1 were stained with anti-HLA-DR mAb (clone:LN3) (Biolegend CA) and analyzed by flow cytometry as described in Materials and Methods. Green lines show staining profiles of HLA-DR.
(TIFF)

S2 Fig Kinetics and dose dependency of mAb 4713 cytotoxicity. **A. Kinetics of cytotoxic effect of mAb 4713 on L428. B. Dose-dependent cytotoxic effect of mAb 4713 on L428.** Target cells (L428) were resuspended at 10^6^/ml in RPMI supplemented with 2%FCS. mAb was added at 3 μg/ml (A) or the indicated concentrations (B) for the indicated periods (A) or 120 min (B). The percentage of alive cells and %cytotoxicity were determined by dye exclusion test triplicate.
(TIFF)

S3 Fig Caspase-3-independent cytotoxicity of mAb 4713. **A. Western blotting analysis.** Activation (cleavage) of caspase-3 was detected in positive control Jurkat cells treated with cytochrome C, but not L428 cells treated with mAb 4713. We performed this experiment using Apoptosis Marker: Cleaved Caspase-3 (Asp175) Western Detection kit (Cell Signaling Technology, MA)**. B. Flow cytometric analysis.** After treatment with anti-Fas mAb or mAb 4713, target cells (Jurkat and L428) were stained with cleaved caspase-3 (Asp175)-specific antibody (Cell Signaling) and analyzed by flow cytometry.
(TIFF)

S4 Fig Mitochondrial membrane depolarization was not induced by mAb 4713. L428 cells were incubated with 1 μg/ml anti-Fas mAb for 8h, 3 μg/ml mAb 4713 for 30 min, or 50M CCCP for 5h, followed by staining with Mito Probe JC-1 (Abcam). JC-1 red fluorescence was analyzed by flow cytometry.
(TIFF)

S5 Fig Cellular Reactive Oxygen Species (ROS) was not produced by mAb 4713-induced cell death. L428 cells were labeled with 20μ M 2’, 7’-dichlorofluorescin diacetate (DCFDA) and incubated with 3μg/ml of mAb 4713 for 30 min or 0.5M of tert-butyl hydrogen peroxide (TBHP9 for 5h, then analyzed by flow cytometry. ROS was not produced by incubation with mAb 4713.
(TIFF)

S6 Fig Scanning Microscopy findings. MAb RE2 (anti-mouse pan MHC class I mAb)-induced giant pore on the surface of target T cell within 5 min. To prepare the cells for observation with a scanning electron microscope, MS-S2 cells were incubated with RE2 mAb (anti-pan MHC class I mAb) at 37°C for 5 min and then washed with and resuspended in PBS containing 2% FCS. The suspension was fixed with 10 vol of 1% glutaraldehyde in 0.1 M cacodylate buffer (pH 7.3) at 4°C for 2h. Fixed cells were mounted on electric conductive double sided tape (Nisshin EM, Tokyo, Japan) coated with gold-palladium coating system (Polaron, England), and they were examined by a scanning electron scope (model S-430; Hitachi Ltd., Tokyo, Japan). Cells: Helper T cell clone MS-S2 have been established from C3H mouse as previously described [11].
(TIFF)
