# Supplementary figures and images for: Establishment of a Therapeutic Anti-Pan HLA-Class II Monoclonal Antibody That Directly Induces Lymphoma Cell Death via Large Pore Formation
Source: PLoS One. 2016 Mar 30;11(3):e0150496. doi: 10.1371/journal.pone.0150496 (PMC4814124; doi:10.1371/journal.pone.0150496)

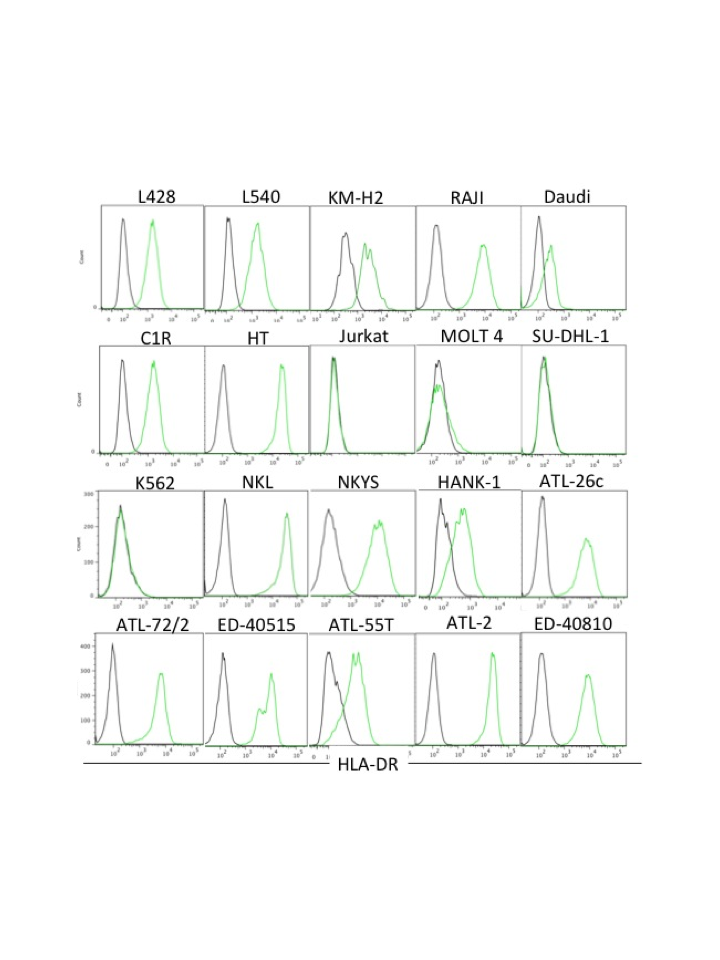

Supplement: S1 Fig — Various cell lines indicate in Table 1 were stained with anti-HLA-DR mAb (clone:LN3) (Biolegend CA) and analyzed by flow cytometry as described in Materials and Methods. Green lines show staining profiles of HLA-DR. (TIFF) [file pone.0150496.s001.tiff]

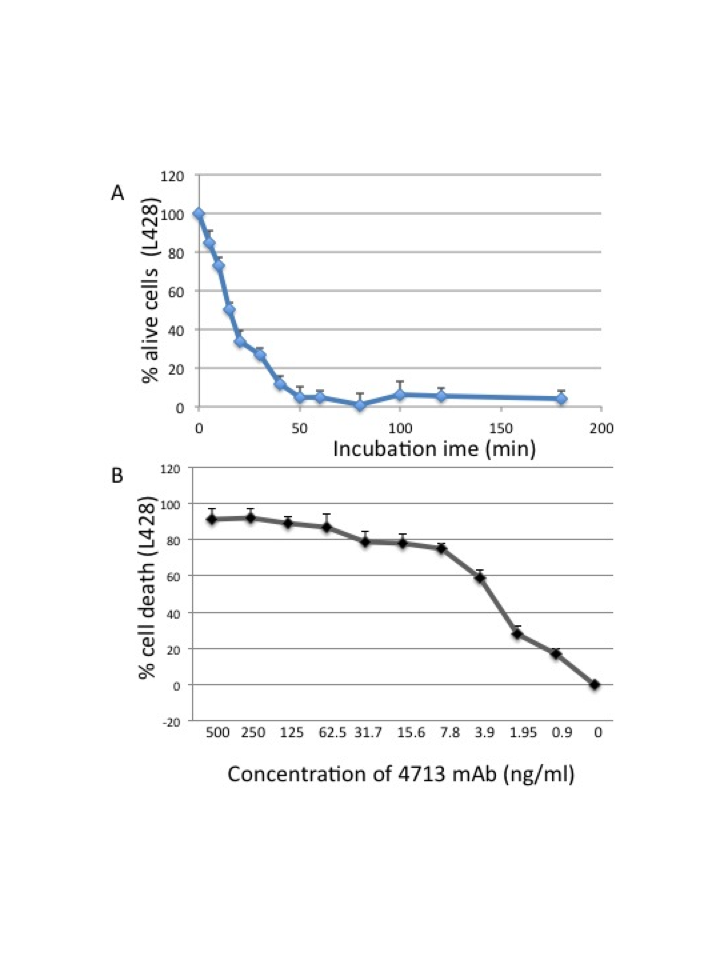

Supplement: S2 Fig — A. Kinetics of cytotoxic effect of mAb 4713 on L428. B. Dose-dependent cytotoxic effect of mAb 4713 on L428. Target cells (L428) were resuspended at 106/ml in RPMI supplemented with 2%FCS. mAb was added at 3 μg/ml (A) or the indicated concentrations (B) for the indicated periods (A) or 120 min (B). The percentage of alive cells and %cytotoxicity were determined by dye exclusion test triplicate. (TIFF) [file pone.0150496.s002.tiff]

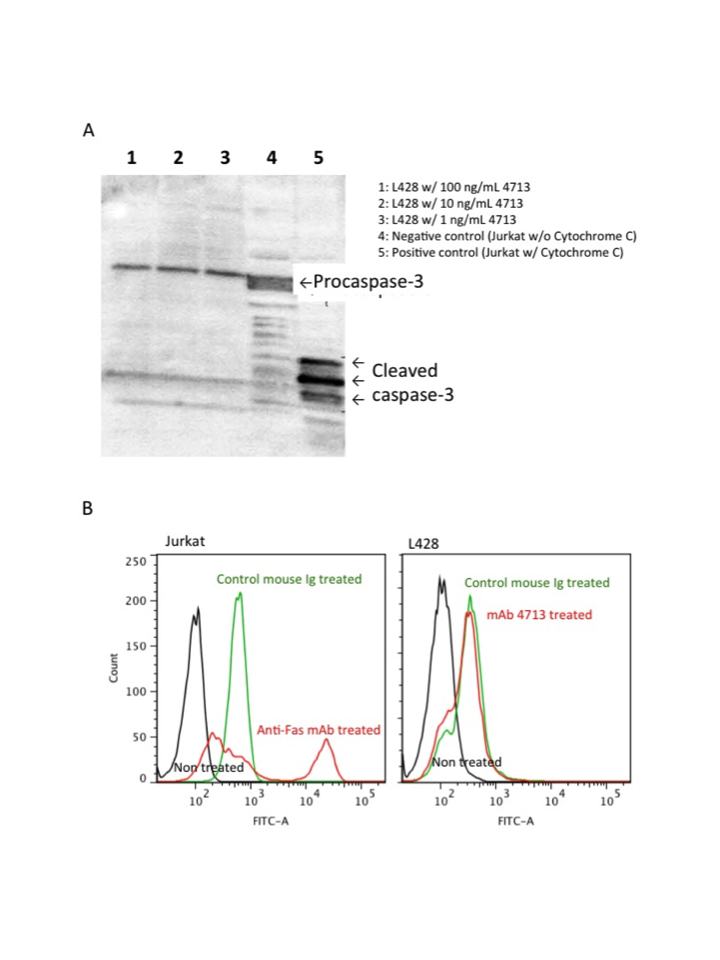

Supplement: S3 Fig — A. Western blotting analysis. Activation (cleavage) of caspase-3 was detected in positive control Jurkat cells treated with cytochrome C, but not L428 cells treated with mAb 4713. We performed this experiment using Apoptosis Marker: Cleaved Caspase-3 (Asp175) Western Detection kit (Cell Signaling Technology, MA). B. Flow cytometric analysis. After treatment with anti-Fas mAb or mAb 4713, target cells (Jurkat and L428) were stained with cleaved caspase-3 (Asp175)-specific antibody (Cell Signaling) and analyzed by flow cytometry. (TIFF) [file pone.0150496.s003.tiff]

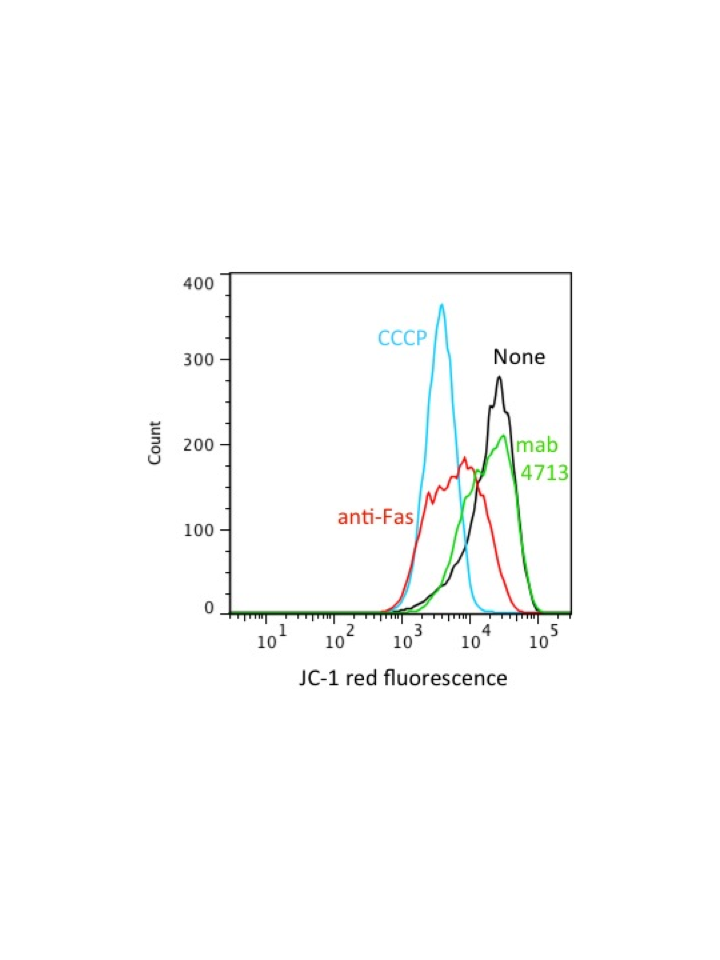

Supplement: S4 Fig — L428 cells were incubated with 1 μg/ml anti-Fas mAb for 8h, 3 μg/ml mAb 4713 for 30 min, or 50M CCCP for 5h, followed by staining with Mito Probe JC-1 (Abcam). JC-1 red fluorescence was analyzed by flow cytometry. (TIFF) [file pone.0150496.s004.tiff]

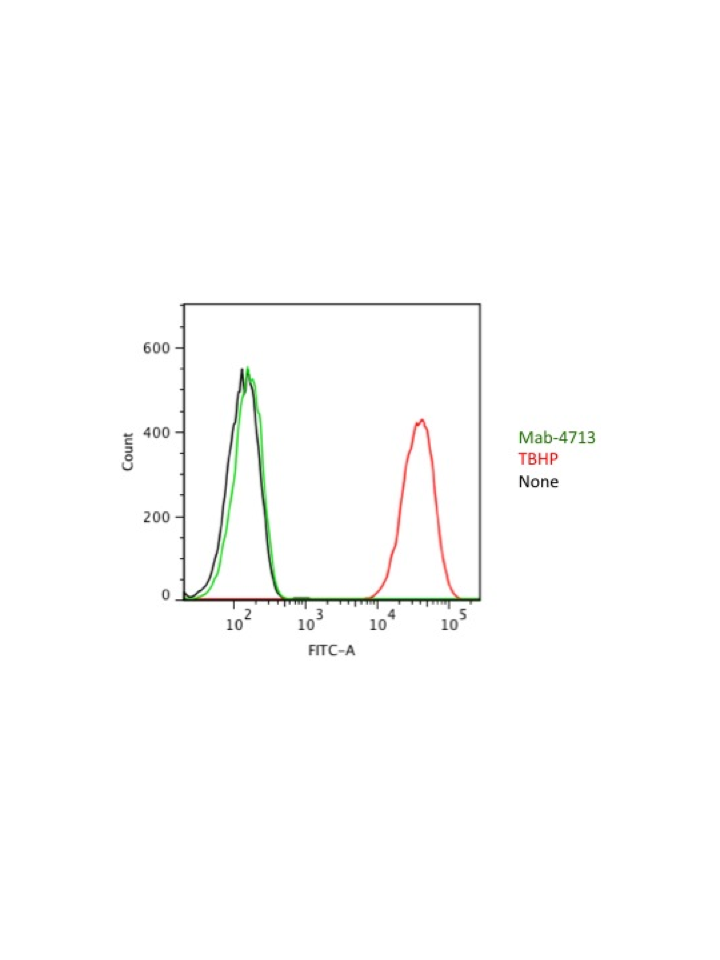

Supplement: S5 Fig — L428 cells were labeled with 20μ M 2’, 7’-dichlorofluorescin diacetate (DCFDA) and incubated with 3μg/ml of mAb 4713 for 30 min or 0.5M of tert-butyl hydrogen peroxide (TBHP9 for 5h, then analyzed by flow cytometry. ROS was not produced by incubation with mAb 4713. (TIFF) [file pone.0150496.s005.tiff]

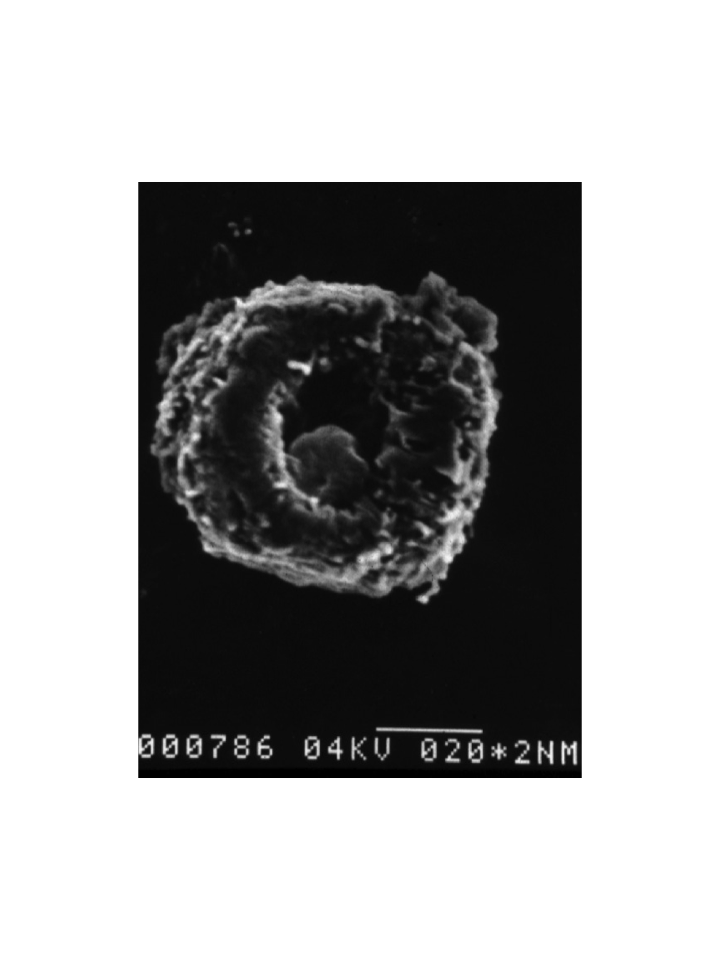

Supplement: S6 Fig — MAb RE2 (anti-mouse pan MHC class I mAb)-induced giant pore on the surface of target T cell within 5 min. To prepare the cells for observation with a scanning electron microscope, MS-S2 cells were incubated with RE2 mAb (anti-pan MHC class I mAb) at 37°C for 5 min and then washed with and resuspended in PBS containing 2% FCS. The suspension was fixed with 10 vol of 1% glutaraldehyde in 0.1 M cacodylate buffer (pH 7.3) at 4°C for 2h. Fixed cells were mounted on electric conductive double sided tape (Nisshin EM, Tokyo, Japan) coated with gold-palladium coating system (Polaron, England), and they were examined by a scanning electron scope (model S-430; Hitachi Ltd., Tokyo, Japan). Cells: Helper T cell clone MS-S2 have been established from C3H mouse as previously described [11]. (TIFF) [file pone.0150496.s006.tiff]
